# Single-cell multi-omics analysis identifies metabolism-linked epigenetic reprogramming as a driver of therapy-resistant medulloblastoma

**DOI:** 10.21203/rs.3.rs-5522707/v1

**Published:** 2024-12-13

**Authors:** Bethany Veo, Dong Wang, John DeSisto, Angela Pierce, Rajeev Vibhakar

**Affiliations:** 1 Department of Pediatrics, University of Colorado Anschutz Medical Campus, Aurora, CO, USA; 2 Morgan Adams Foundation Pediatric Brain Tumor Research Program, Children’s Hospital Colorado, Aurora, CO, USA; 3 University of Colorado Cancer Center, Aurora, CO, USA

## Abstract

Medulloblastoma (MB) is the most prevalent malignant brain tumor in children, exhibiting clinical and genomic heterogeneity. Of the four major subgroups, Group 3 tumors (MYC-MB), display high levels of MYC and metastasis rates. Despite treatment with surgery, radiation, and chemotherapy, patients with Group 3 MB are more likely to develop aggressive recurrent tumors with poor survival. To examine resistance mechanisms, single nuclei multiome analysis of matched primary and recurrent tumors was performed in this study. A persistent progenitor population supporting resistance to therapy was identified. Additionally, distinct chromatin landscapes are linked to altered transcription and correspond to metabolic reprogramming. *In vivo* modeling of radiation resistance resolves similar chromatin-based metabolic reprogramming focused on wild-type isocitrate dehydrogenase (IDH1) activity. IDH1 inhibition reverses resistance-mediated chromatin changes and enables radiation re-sensitization. Ultimately, these findings demonstrate the efficacy of single-cell multiome analysis in elucidating resistance mechanisms and identifying novel target pathways for MYC-driven medulloblastoma.

## Introduction

Medulloblastoma (MB) is the prevailing malignant brain tumor in children, accounting for 20% of all pediatric brain tumors^1,2^. MB is clinically heterogeneous, with varying prognoses depending on the molecular subgroup. Group 3 tumors with *Myc* amplification exhibit higher rates of metastasis and recurrence^3,4^. Treatments for these high-risk patients include craniospinal irradiation (36Gy) after surgical resection; however, most patients relapse within 1 year^1,5,6^. Despite this, radiation remains the primary therapeutic option for high-risk MB patients, culminating in <5% long-term survival^1,7–9^.

Tumor heterogeneity driven by clonal evolution and tumor-initiating cell diversity is thought to contribute to therapy failure^10–12^. Recent single-cell RNA-seq (scRNA-seq) studies have validated the intra- and inter-tumoral heterogeneity of medulloblastoma^13–15^. We previously identified a stem-like population in Group 3 tumors with a gene signature that predicts poor outcomes^13^. Although, MB molecular subtypes present differing relapse patterns and evolution of chromosomal changes, new genetic events are rare in Group 3 medulloblastoma^16^.

Cellular plasticity is another major driver of therapy resistance. Cancer cells can alter their lineage by reverting to a stem-like state, evading therapies targeting the original lineage-directed survival programs. A MB disease progression model of relapse highlighted this phenomenon, noting a population of MB stem-like cancer cells averted standard therapies by increasing self-renewal^17^. Although many genomic aberrations have been associated with lineage plasticity, few tumors exhibit these alterations, suggesting that resistance due to lineage plasticity occurs through unidentified mechanisms. Moreover, epigenetic reprogramming impacts cellular lineage decisions and cell state changes that reduce responsiveness to therapy^18–22^. Considering that no current therapies effectively target lineage plasticity-driven resistance in tumors, an urgent need exists to identify key lineage plasticity modifiers as potential therapeutic targets.

In this study, single-cell multiome sequencing of matched primary and recurrent MB was performed to explore the heterogeneous cell populations response to therapy. Our data identifies cell populations that demonstrate plasticity and chromatin remodeling during resistance to therapy. Further, we determined chromatin remodeling reprograms the metabolic state demonstrating the potential for phenotypic targeting of a resistant cell population by disrupting lineage directed plasticity.

## Results

### scMultiome-seq profiling of matched relapsed and primary MB tumors

**Extended Data Figure 1.**
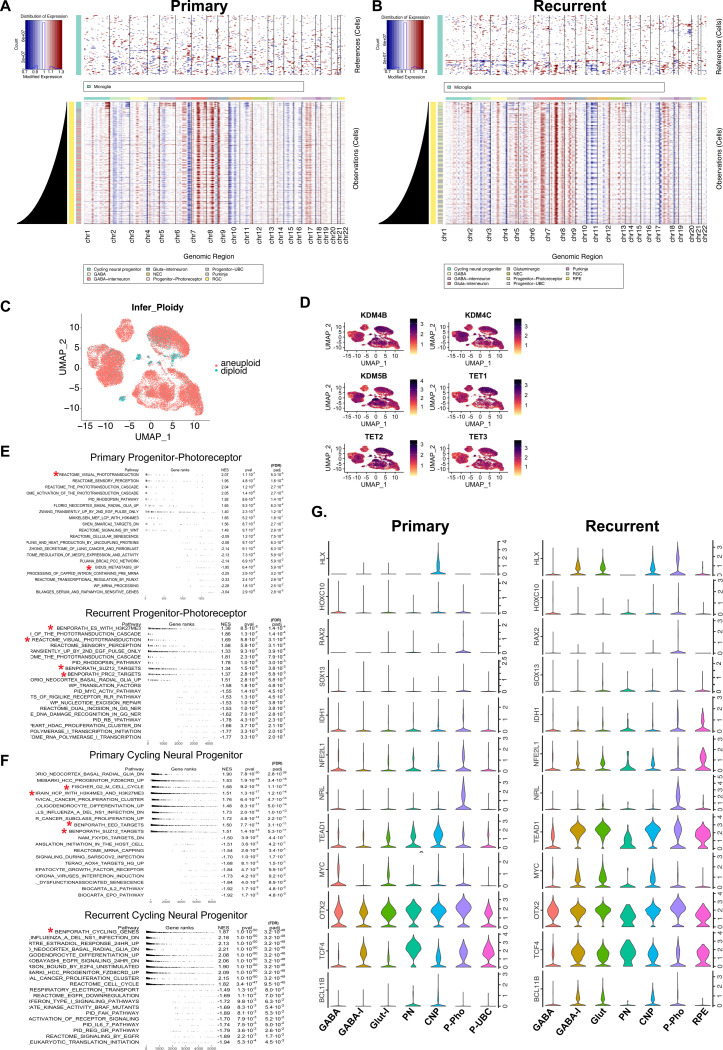
Recurrent populations are enriched for metabolic and chromatin modifiers. **A.** Hierarchical heatmap showing copy-number variation in primary tumors, **B.** Recurrent tumors inferred by InferCNV. (Above) Reference non-tumor cells. (Below) Copy number variations detected are consistent with Group 3 and Group 4 amplification. **C.** UMAP representation of cells designated aneuploid or diploid based on inferred CNV analysis. **D**. UMAP representation of marker gene expression for α-KG responsive enzymes. **E**. GSEA enrichment plots comparing primary and recurrent tumor populations in the progenitor-photoreceptor cluster and **F**. cycling neural progenitor cluster. NES and FDR values are indicated. **G.** Gene expression violin plots of key genes within the progenitor_photoreceptor gene signature in primary tumor compared to recurrent tumors by cluster. GABA (GABAergic), GABA-I (GABAergic-Interneuron), Glut-I (Glutamatergic-Interneuron), Glut (Glutamatergic), PN (Purkinje), CNP (Cycling Neural Progenitor), P-Photo (Progenitor-photoreceptor), P-UBC (Progenitor-UBC), RPE (Retinal Pigment Epithelium).

**Figure 1.**
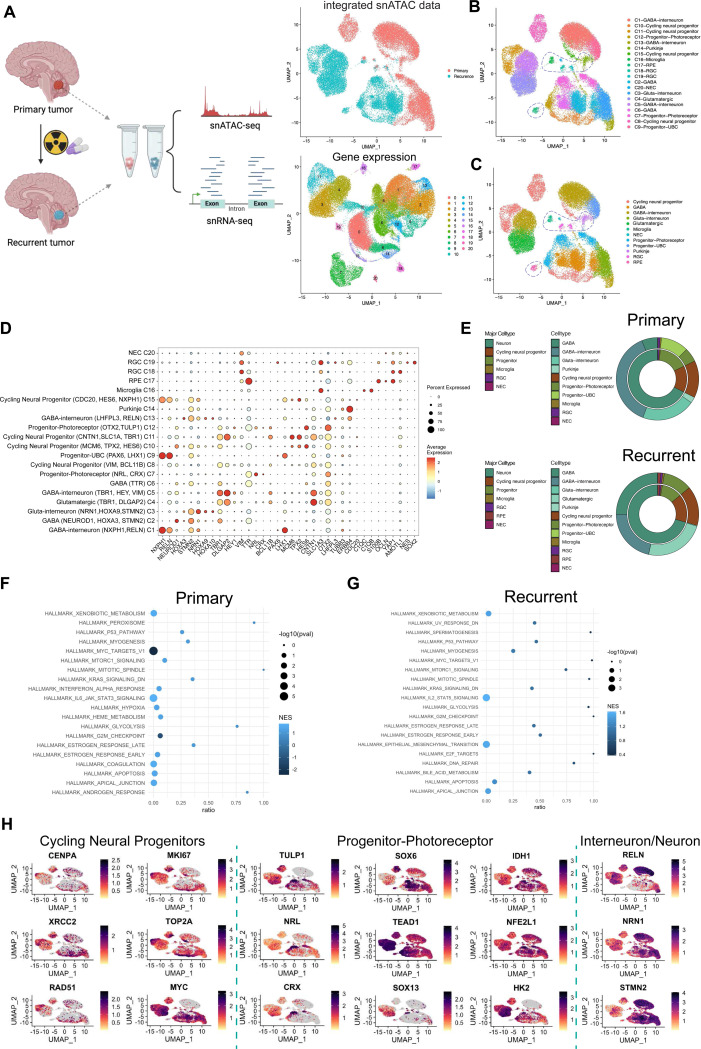
scMultiome-sequencing of matched primary and recurrent medulloblastoma. **A**. Schematic representation of matched primary and recurrent tumor processing for scMultiome. Joint Uniform Manifold and Projection (UMAPs) were generated using principal component analysis (PCA) dimensional reduction for gene expression and Latent semantic indexing (LSI) indexing for DNA accessibility. (Below) UMAP visualization of scRNAseq data from patient primary (*n=3*) and recurrent tumor samples (*n=3*). Coloring based on clustering. (Above) UMAP visualization of integrated scATAC and scRNA-seq datasets. Color designates primary cells (pink) and recurrent cells (turquoise). Total nuclei count=50,184. **B**. Cell state designation of 20 clusters based on FindClusters. Non-malignant, diploid cells are circled. **C**. Merged cell states of 12 major clusters. **D**. Gene expression marker dot plot. Color indicates average expression and dot size refers to the percentage of cells within the cluster expressing the gene. **E**. Cell proportions in 12 major clusters and 20 minor clusters. Colored by cell type. **F**. fGSEA dot plot of enriched paths in primary and **G**. recurrent tumor populations. -Log_10_(Pval) and normalized enrichment score is shown **H**. Representative marker gene expression from progenitor-photoreceptor cell state, cycling neural progenitor cell state, and Interneuron/Neuron cell state. *See also* Extended data Figure 1.

ScMulti-omic sequencing of patient-matched primary and relapsed MB tumors was performed to examine the epigenetic profile of recurrent tumor samples. Single nuclei were isolated from three pairs of primary and recurrent MB tumor samples. After stringent quality control and doublet removal, 50,184 nuclei were profiled for sequencing. Unsupervised clustering identified 20 clusters with initial cell-type annotations based on differentially expressed genes (DEG; Figure 1A). ScATAC and scRNA-seq data were integrated to identify cell states based on transcriptomic and chromatin accessibility changes; clustering retained 20 cell-state identities, which were further consolidated into 12 major cell groups (Figure 1B, C). Cell-state annotation was based on gene expression patterns from early cerebellar development and prior single-cell MB patient analysis^23–30^. Normal diploid cells were identified by inferCNV analysis (Extended Data Figure 1) and cell identification markers for microglia (*C1qb*), radial glial cells (RGC) (*AQP4, SOX2, NES*, and *AMOT1L*), retinal pigment epithelial cells (RPE), neuronal epithelial cells (NEC) (*NES, YAP1, FAT4*, and *OCLN*), and Purkinje cells (Figure 1B, C). Malignant cells comprised of cycling neural progenitors defined by *MKI67, MYC*, and *TOP2A*. Non-cycling neural progenitors expressed genes from the subventricular zone of the Rhombic Lip and early photoreceptor (*NRL, CRX, OTX2,* and *TULP1*) and unipolar brush cell (UBC) lineages (*PAX6* and *EOMES*; Figure 1D). Additionally, malignant cells expressing *TBR1, NEUROD1*, and *STMN2* resembled GABAergic and glutamatergic interneurons and neurons (Figure 1D). The proportion of the progenitor_photoreceptor cell state and cycling neural progenitor cell states increased by 2-fold in recurrences, whereas the proportion of more differentiated neuron-like cells were relatively unchanged (Figure 1E).

Comparison of the gene expression differences between recurrent tumor populations and primary tumors by fGSEA revealed an enhanced association with metabolism, stemness, the epithelial-mesenchymal transition (EMT), and DNA repair (Figure 1F, G). Additional profiling of the progenitor_photoreceptor populations in primary versus recurrent tumors identified enrichment of genes normally associated with high CpG density and H3K4me2/3 and H3K27me3 alterations in recurrences (Figure 1F, G and Extended Data Figure 1). Indeed, expanded expression of key determinants of neural progenitor fate and maintenance (*SOX6, TCF4*, and *TEAD1*) was observed primarily in progenitor_photoreceptor clusters and elevated in recurrent samples (Figure 1H). The upregulation of genes that modulate metabolic responses and energy production, including *IDH1, HK2, HSPH1, GLS*, and *NFE2L1*, largely overlapped with the recurrent progenitor_photoreceptor cluster. This subpopulation was also enriched in α-ketoglutarate-responsive histone demethylases (Extended Data Figure 1). These genes were used to define the progenitor_photoreceptor populations based on a gene signature amplified in recurrent tumor samples (Figure 1H, Extended Data Figure 1).

### Chromatin restructuring in recurrent samples promote a switch in network programming

**Figure 2.**
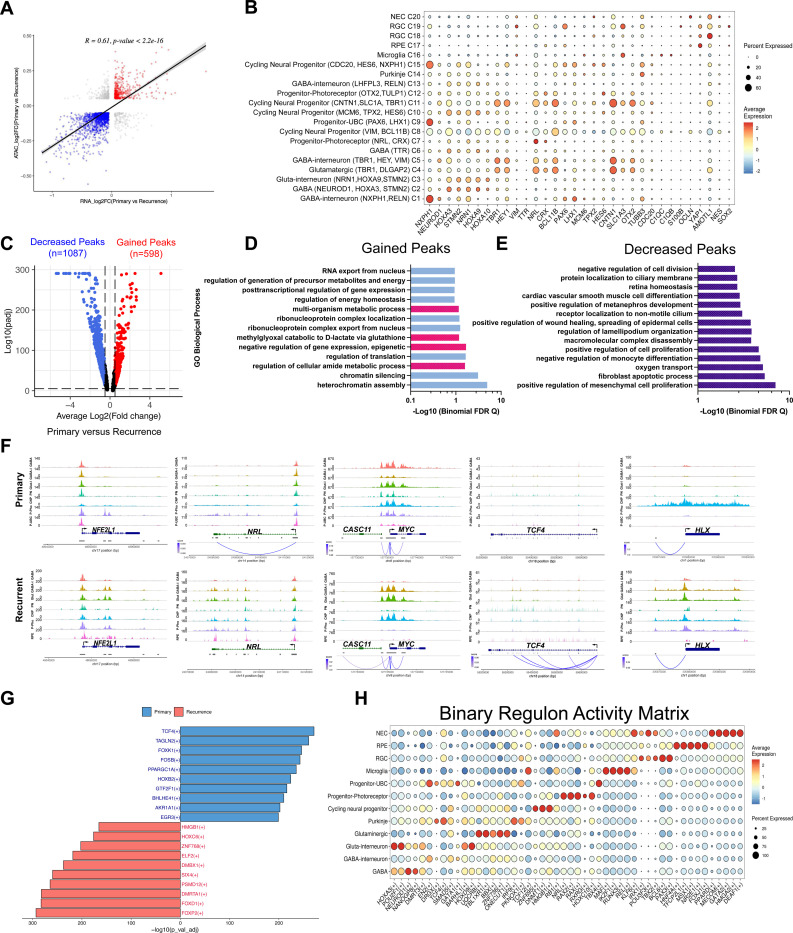
Altered chromatin accessibility in recurrent tumors modulates network programming **A.** Two-tailed Pearson’s correlation analysis of peak accessibility (ATAC-seq) and DEGs (RNA-seq). Log_2_FC in primary vs recurrent tumors is plotted. **B**. Chromatin accessibility matrix with gene expression markers by cell state. Color indicates gene accessibility and dot size refers to the percent of cells in the cluster **C**. Differential accessibility of peaks in primary vs recurrent tumor cells. Volcano plot of the average Log_2_FC of reads/peak against -Log_10_(padj) **D**. Functional enrichment of gained peaks as determined by GREAT v4.0.4. Metabolic and epigenetic associations are highlighted in pink. − Log10(Binomial FDR Q) is reported **E**. Functional enrichment of decreased peaks. − Log10(Binomial FDR Q) is reported. **F**. Peak accessibility profiles by cell state with proximal gene linkages for genes (NFE2L1, NRL, MYC, TCF4, and HLX) representative of the changing cell states in primary and recurrent tumor. The height and color intensity of linkages indicate accessibility score. GABA (GABAergic), GABA-I (GABAergic-Interneuron), Glut-I (Glutamatergic-Interneuron), Glut (Glutamatergic), PN (Purkinje), CNP (Cycling Neural Progenitor), P-Photo (Progenitor-photoreceptor), P-UBC (Progenitor-UBC), RPE (Retinal Pigment Epithelium). **G**. Top ten gene regulatory networks in primary compared to recurrence. -Log_10_(Adj pVal) is reported. **H**. Dot plot of the top active gene regulatory networks by cell state. P<0.01.

To compare the chromatin profiles of the primary and recurrent tumors, InferCNV detected prior and new cytogenetic abnormalities at recurrence. Heatmaps show a gain of Ch7 and 8 in primary tumors, consistent with known Group 3 and Group 4 amplifications^3,31^. The matched recurrent tumors maintained the gain of Ch7, whereas no new major alterations were detected (Extended Data Figure1). Comparison between snATAC and snGEX patterns revealed that shifting chromatin accessibility was positively correlated with altered gene expression (Figure 2A). Furthermore, the chromatin accessibility matrix showed open chromatin regions associated with the cell-state marker genes identified (Figure 1B), further demonstrating the correspondence between transcriptomic and chromatin structure (Figure 2B). Gains were observed in 598 peaks and a decrease or loss in 1087 peaks in recurrent tumors compared to primary tumor samples (Figure 2C). The proximal cis-regulatory regions associated with gained peaks were predominately associated with the regulation of chromatin silencing and metabolic program regulation (cellular amide and methylglyoxal conversion to D-lactate via glutathione) (Figure 2D). The decreased peaks were associated with oxygen transport and differentiation (Figure 2E). These data suggest, recurrent tumors show signs of chromatin restructuring and utilize alternative metabolic pathways to enable survival with the downregulation of oxygen availability (i.e., hypoxia) and differentiation programs.

Gene-specific peak profiles were evaluated to determine the differences in gene accessibility profiles in primary and recurrent tumors based on cell state. Open chromatin regions in primary tumors indicated cell-state specificity of peaks for *NFE2L1, TCF4*, and *HLX*. Chromatin accessibility increased to all cell states and condensed in specific cell states in recurrent tumors, modulating gene expression (Figure 2F). Additionally, proximal gene linkages between regulatory elements and gene promoters were established for *TCF4* and *MYC*, manipulating gene expression by altering the chromatin landscape.

To then understand how the gene regulatory networks (GRNs) in primary tumors change with respect to recurrence, the integrated snATAC and snGEX data was used to determine the active GRNs. The top GRNs in primary tumors were *TCF4, TAGLN2, FOXK1*, and *BHLHE41*, whereas the top upregulated GRNs in recurrent tumor cells were *FOXP2, FOXD1, SIX4*, and *HMGB1* (Figure 2G). The top ten network regulon activity was mapped to the 12 major cell-states (Figure 2H). Non-cycling progenitor populations showed specific GRN activity with *TBX4, DRGX*, and *EN2* in UBC-linked progenitors, whereas *HOXC10, NRL*, and *RAX* regulons were most active in photoreceptor-linked progenitors. The most active GRNs in the cycling neural progenitors were *HMGB1, ZNF695*, and *DNMT1* (Figure 2H). Analysis of integrated snGEX and snATAC in primary and recurrent tumors suggests that recurrences undergo chromatin restructuring, facilitating a switch in network programming that favors pluripotency and metabolism despite oxygen depletion.

### Trajectory analysis suggests the amplification of the progenitor_photoreceptor lineage in relapsed Grp3-MB

**Figure 3.**
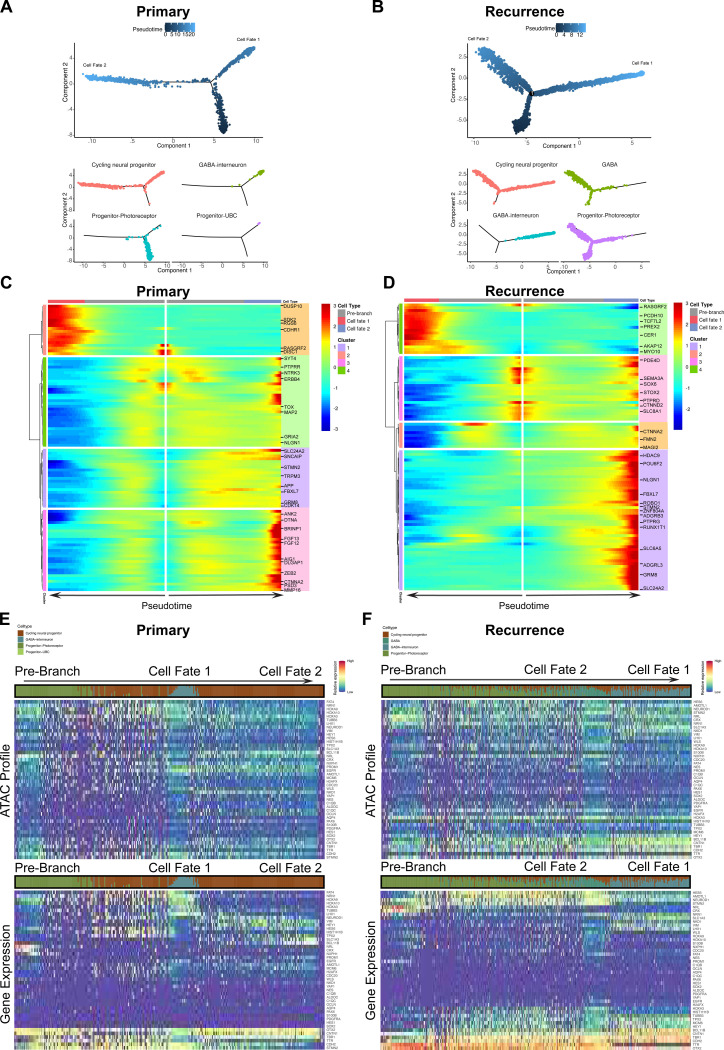
Pseudotime analysis projects cell-state progression in recurrent tumors **A.** Pseudotime ordering of primary tumor cell states (CNP, GABA-I, Pro-photo, Pro-UBC) **B**. Recurrent tumor cell states (CNP, GABA-I, Pro-photo, GABA) on a bifurcated cell trajectory, (above) color scale based on pseudotime, (below) separated and colored by cell state. **C**. Heatmap of hierarchically clustered expression of branch dependent genes based on branched expression analysis modeling (BEAM) for primary, **D**. Recurrence. Direction of pseudotime starts at the pre-branch and follows either cell fate 1 or cell fate 2. **E**. Primary ATAC score (above) and Gene expression (below) along pseudotime trajectory. **F**. Recurrence ATAC score (above) and Gene expression (below) along pseudotime trajectory. Color scale indicates accessibility or relative expression.

To understand the differences in gene expression dynamics as primary tumor cells transition to a resistant state, cell-state progression was mapped along a pseudotemporal trajectory for Grp 3 primary and recurrent tumors. Cell states were ordered along an independent trajectory for primary and recurrent tumor cells (Figure 3). In primary tumors, the pre-branch was occupied by progenitor photoreceptor-like cells that bifurcate into two cell-states: cycling progenitor cells (Cell-state 1) and combined differentiated and cycling cells (Cell-state 2; Figure 3A). Consequently, in recurrences, the early progenitor photoreceptor-like state occupied the pre-branch and contributed to the amplification of cycling progenitor and more differentiated states in Cell-state 2, similar to the primary tumor. However, in Cell-state 1, the progenitor photoreceptor-like cells lead to a less differentiated state (Figure 3B).

**Extended Data Figure 2.**
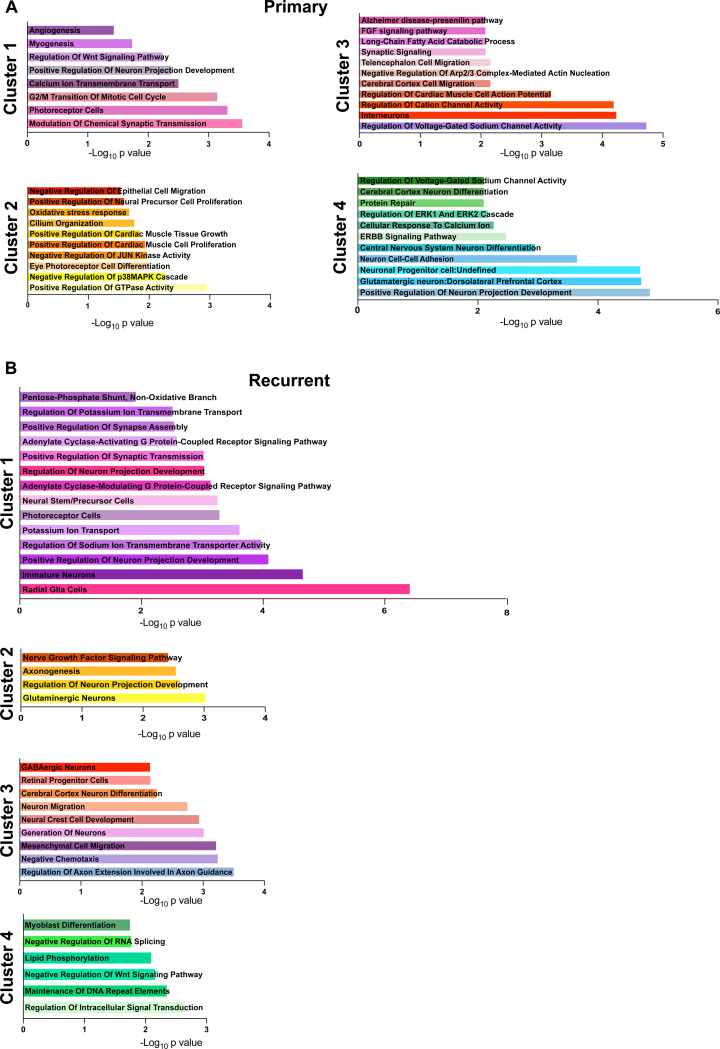
Enrichment analysis of BEAM DEGs. **A.** Enrichr analysis of differential gene expression by cluster in primary BEAM results. **B**. Enrichr analysis of differential gene expression by cluster in recurrent BEAM results. -Log10(PValue) is reported. Color code matches cluster color designation.

Hierarchical clustering of differential expression between individual cell-state branches revealed upregulated expression of *RGS7, TOX, SEMA3A, SOX6*, and *ESRRG* within the recurrent pre-branch (Figure 3C and D). Enrichment analysis suggested that these cells aligned with retinal progenitor cells (RPCs) and promoted a photoreceptor-like state in the later branches (Extended Data Figure 2). Additionally, genes mediating the pentose-phosphate shunt and metabolic processes were amplified, as is often exhibited by neural stem cells (NSCs) and retinal progenitors. In recurrent tumors, this population was heavily maintained at Cell-state 2, whereas in primary tumors, the cells dissipate beyond the branch point.

Next, ATAC and GEX profiles were mapped onto the pseudotime trajectory for the cell-state markers. In primary tumors, marker gene accessibility was succinct in progenitor_photoreceptor and interneuron cells states, whereas in recurrent tumors, chromatin accessibility was open and extended across length of the trajectory (Figure 3E and F). Additionally, the expression of key markers such as *NRL, CRX, LHX1*, and *OTX2* aligned with the progenitor_photoreceptor and interneuron cell states. Recurrences showed amplification and extended *NRL, OTX2*, and *TTR* expression throughout the trajectory. Hence, the progenitor_photoreceptor-like cells are present in both primary and recurrent tumors and represent two cell-states: an early state that resembles multipotent RPCs and a later, more determined photoreceptor-like state.

### Progenitor_Photoreceptor signature maps to relapse MB.

**Figure 4.**
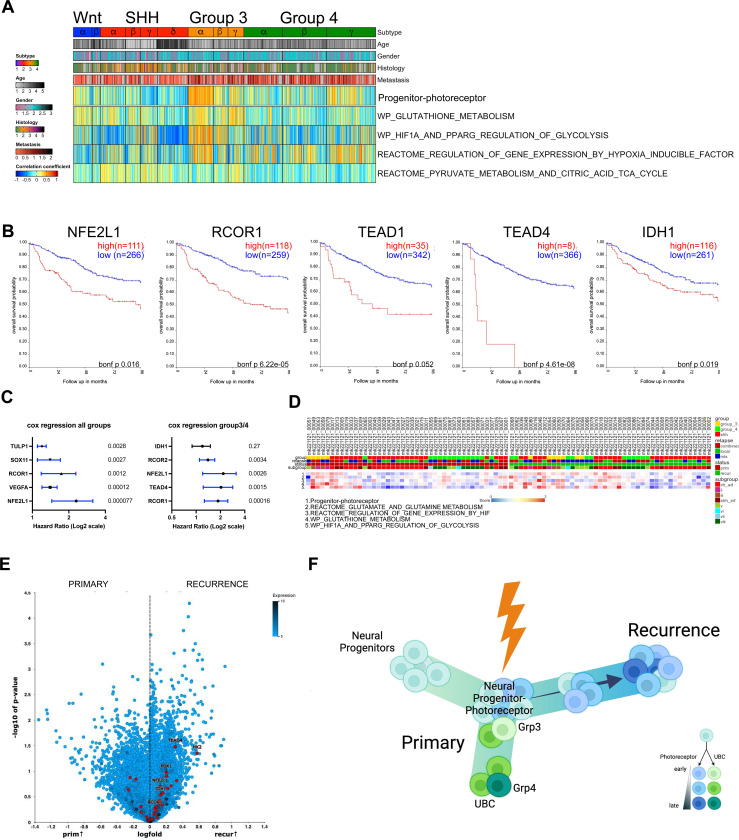
Progenitor-photoreceptor gene signature aligns with Group 3 type tumors and relapsed metastatic lesions. **A.** GSVA of Progenitor-photoreceptor gene signature comparison to the Cavalii data set. **B**. Kaplan-Meier survival curves in Group 3 MB for representative genes in the progenitor-photoreceptor gene signature. Bonferroni p-value is shown. **C**. Cox regression analysis for the Progenitor-photoreceptor gene signature in all MB subgroups. (Right) in Group3/4 MB. Hazard ratio is graphed with p-value on the right. **D**. Gene signature heatmap comparison to the Okonechnikov data set. Samples are split by primary and recurrence. **E**. Gene expression volcano plot of primary vs recurrence from the Okonechnikov data set. Progenitor-photoreceptor gene signature is overlaid in red. **F.** Model of the development of recurrence from a resilient cell resembling the progenitor-photoreceptor population.

To evaluate if the progenitor_photoreceptor signature was present in a larger cohort of patient transcriptomic data, the Cavalli dataset^3^ (RNA-seq of 763 MB tumors) and Korshunov dataset^32^ (84 primary and recurrent MB tumors) were assessed. The photoreceptor_progenitor gene signature was evaluated with glutathione metabolism and hypoxia gene signatures from msigDB, by gene set variation analysis (GSVA). Overall, gene expression overlapped between the photoreceptor_progenitor and metabolic signatures in Group 3 and 4 tumors (Figure 4A). A decreased survival benefit was observed in Group 3 and 4 tumors for several key factors within the progenitor_photoreceptor signature, including *NFE2L1, TEAD1, TEAD4,* and *IDH1* (Figure 4B). Additionally, Cox regression analysis identified a significant hazard ratio for genes within the progenitor_photoreceptor signature in all MB subgroups and in Group 3 and 4 tumors (Figure 4C). Comparison of the primary and recurrent MB subgroups showed the progenitor_photoreceptor gene signature aligned with metabolic and hypoxia-inducible factor (HIF) regulation gene signatures (Figure 4D). Additionally, heightened expression of the photoreceptor_progenitor signature in recurrences and relapses with metastatic lesions, was observed (Figure 4D and 4E). This data suggests a cell-state change in early progenitor cells from primary to recurrence may be specified by gene signatures modulating metabolic and pluripotency programs, often observed in normal retinal progenitor cells and quiescent adult NSCs (Figure 4F).

### Photoreceptor_progenitor populations persist in radiation resistance murine models

**Figure 5.**
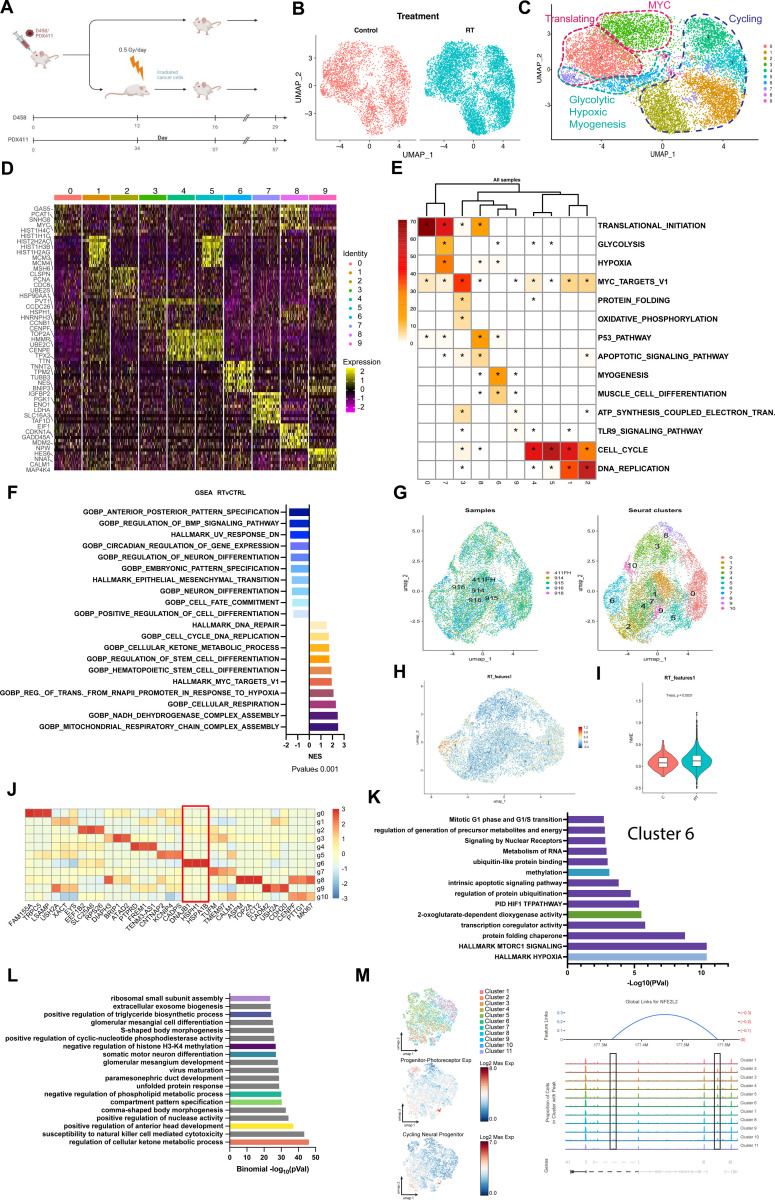
In vivo models of radiation resistance. **A.** Schematic of radiation resistance model development. **B**. UMAP visualization of control tumors(n=2) and radiation resistant tumors (n=4). **C**. Color designation is based on clustering. Cell grouping of clusters is based on ORA GSEA **D**. Heatmap of top DEGs by cluster. **E**. Over-representation analysis (ORA) heatmap based on individual clusters. **F**. GSEA comparing radiation resistant tumors to control. P<0.001. NES is plotted. **G**. UMAP visualization of PDX411 radiation resistant tumors and 411FH control (Hovestadt) **H**. Progenitor-photoreceptor gene signature mapped to Cluster 6. **I**. Violin plot of Progenitor-photoreceptor gene signature expression in radiation resistant tumor compared to control. P<0.0031. **J**. Heatmap of top DEGs by cluster. **K**. Gene ontology analysis of cluster 6. -Log_10_(Pval) is reported. **L**. Functional enrichment of gained peaks. Metabolic and epigenetic associations are highlighted in color. -Log_10_(Binomial FDR Q) is reported. **M**. UMAP visualization of ATAC-seq clusters. The progenitor-photoreceptor gene signature and cycling neural progenitor signature are mapped. (Left) Representative peak profile for NFE2L2 by cluster. Link arc plot is shown between peaks -Log_10_(Pval)=9.43, correlation = 0.28. Peaks are highlighted in black boxes. *See also* Extended Figure 3.

To explore the development of recurrence in Group 3 MB, recurrence after radiation exposure was modeled *in vivo*. Group 3 mouse orthotopic xenografts were created using the D458 cell line and MED411 PDX. Tumors were exposed to 0.5Gy/day for 5 days to model fractionated radiation (Figure 5A). Tumors responded to radiation but subsequently recurred after 3 weeks. Tumors were recovered at the endpoint and subjected to scRNA-seq (D458) and scMultiome-seq (MED411).

Within the D458RT (radiation-treated) model, Seurat clustering analysis identified ten distinct clusters in the D458 dataset (Figure 5B and C). Enrichment analysis classified clusters similar to the patient samples. Clusters 1/2/4/5 were associated with cell cycle and DNA replication, Clusters 0/7 were associated with translation, Cluster 3 *MYC* expression, and Clusters 6/7/8 with glycolysis and hypoxia (Figure 5C, D, E). In D458RT tumors, metabolic programs mediating cellular respiration, modulation of stem cell differentiation, and DNA repair were enhanced compared to radiation-naive tumors. Negative enrichment was observed for differentiation programs and cell fate commitment (Figure 5F).

**Extended Data Figure 3.**
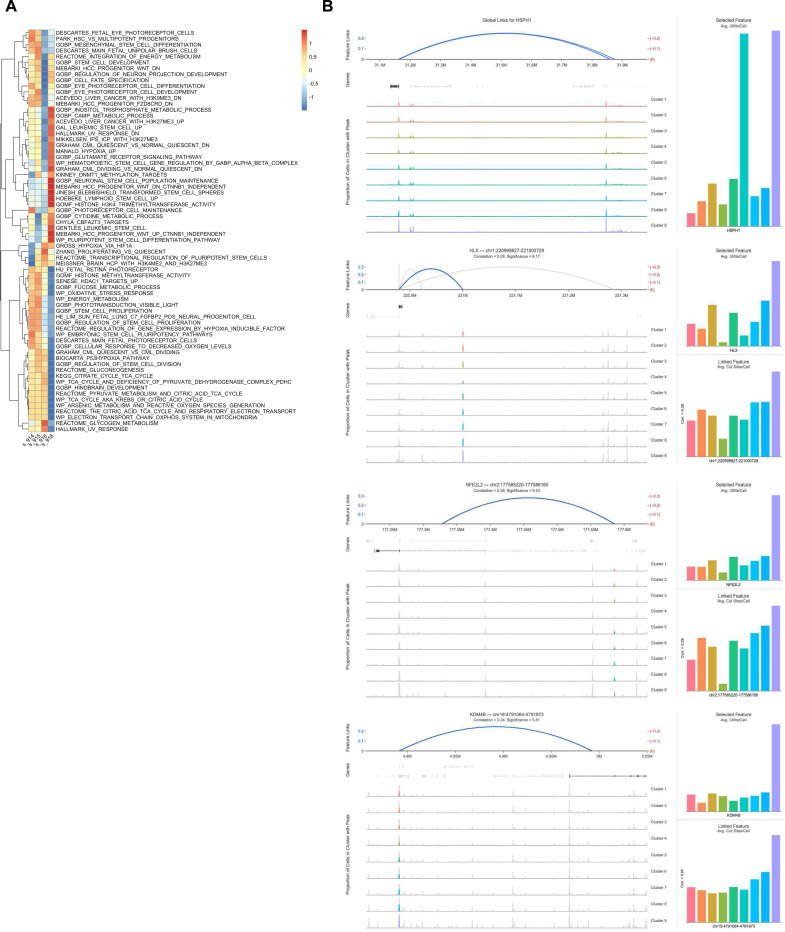
scMultiome analysis of PDX411 radiation resistant tumors display metabolic and pluripotency gene linkages. **A.** Hierarchical clustering heatmap comparing non-IR treated (918) and IR-resistant (914,915,916) against metabolic and pluripotency gene sets. **B**. Gene feature linkage and accessibility for *HSPH1, HLX, NFE2L2*, and *KDM4B* based off snATAC-seq of PDX411.

Seurat analysis of MED411 PDX control and RT-exposed tumors revealed 11 clusters (Figure 5G). The progenitor_photoreceptor gene signature was mapped to Cluster 6, comprising MED411RT (radiation-resistant) cells (Figure 5H). The progenitor_photoreceptor gene signature was significantly upregulated in MED411RT tumors compared to control (Figure 5I). The top expressed genes in Cluster 6 were *DNAJB1, HSPH1*, and *HSPA1B*. GSVA enrichment analysis of Cluster 6 revealed enhanced metabolic and hypoxic pathways and altered 2-oxoglutarate dioxygenase (α-KG) activity (Figure 5J, K, Extended Data Figure 3). Genes proximal to ATAC peaks were associated with metabolic processes, pattern specification, and chromatin modifications, similar to peak locations in recurrent patient samples (Figure 5L). Additionally, ATAC peak profiles for target genes in patient recurrent tumor samples, showed proximal gene linkages in clusters mediating the progenitor_photoreceptor signature and cycling progenitors for regulators such as *NFE2L2, HLX, HSPH1*, and *KDM4B* (Figure 5M, Extended Data Figure 3). This further correlated chromatin restructuring in progenitor_photoreceptor populations with enhanced radiation resistance. Moreover, in both (D458, MED411) radiation-resistant models, we observed gene expression and peak association with genes that mediate metabolic processes, specifically 2-oxoglutarate dioxygenase activity, cellular respiration, and metabolite and energy generation. A decrease in neuronal differentiation programs and an increase in the regulation of stemness differentiation programs and epigenetic modulators that affect histone methylation were observed. These results suggest that limiting conversion of energy sources may impact resistance.

### Disruption of IDH1 activity suppresses radiation resistance and MB proliferation by inhibiting reductive glutamate carboxylation

**Extended Data Figure 4.**
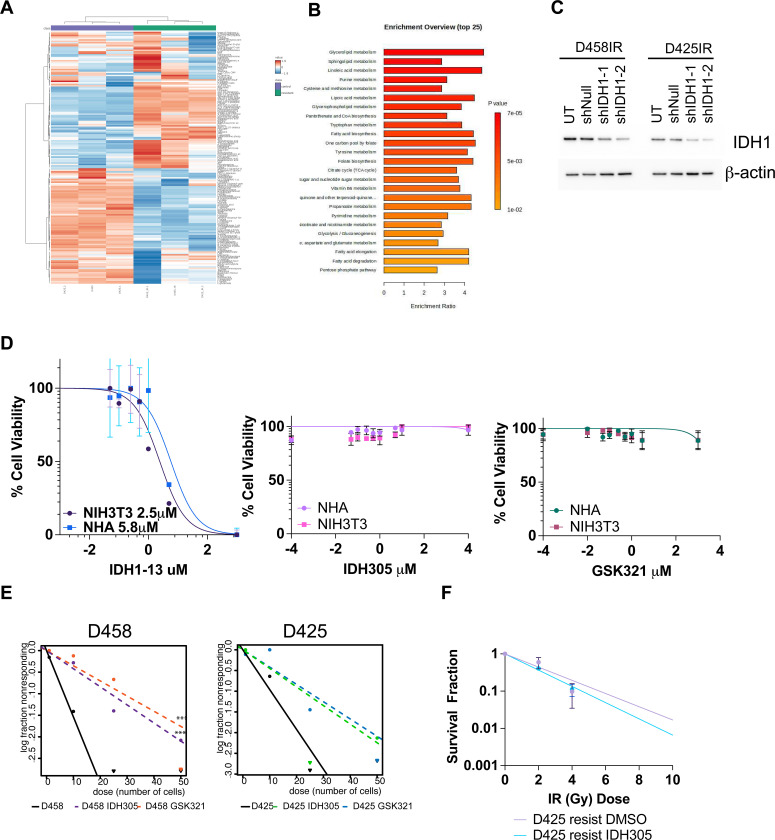
Suppression of IDH1 activity in in vitro radiation resistant models. **A.** GC-MS hierarchical clustering heatmap between radiation resistant (D425IR) and sensitive (D425) cells. Measured using Euclidean distance. **B**. Summary Over Representation Analysis (ORA) in D425IR compared to D425. Hypergeometric distribution test was used. **C**. Western blot validation of shIDH1 knockdown in D458IR and D425IR cells. Two independent shIDH1(TRCN0000027249, TRCN0000027253) constructs were used to deplete IDH1. shNull construct was used as a non-targeting control. **D**. IC50 drug dose curves of IDH305, GSK321, and IDH1–13 on NHA and NIH3T3 cells. **E**. ELDA on parent cell lines D458 and D425 in the presence of IDH305 or GSK321. Log-fraction plot of the limiting dilution model, where the slope of the line is the log-active cell fraction. Chi-square test; ***,p<0.001. **F**. Survival fraction plot of D425IR cells treated with DMSO or IDH305. N=3. Two-way Anova.

**Figure 6.**
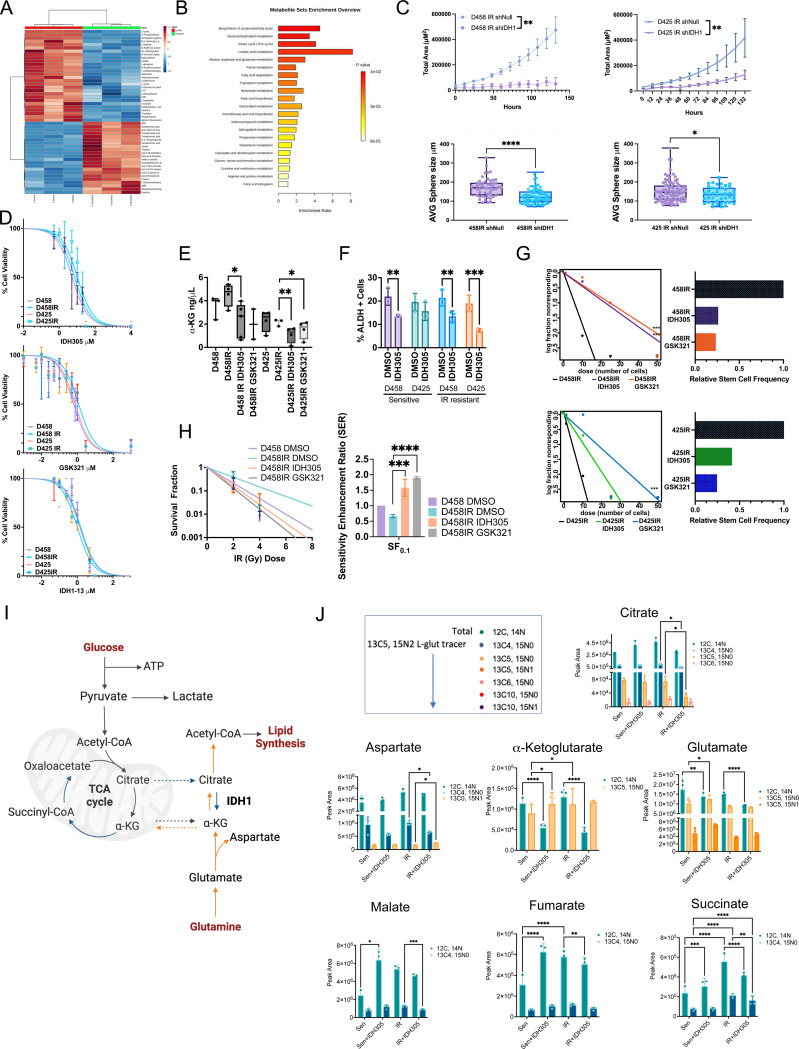
Suppression of IDH1 inhibits proliferation and metabolic rewiring. **A**. Hierarchical clustering heatmap between radiation resistant (D458IR) and sensitive (D458) cells. Measured using Euclidean distance. **B**. Summary Over Representation Analysis (ORA). Hypergeometric distribution test was used. **C**. (Above) Neurosphere assay growth in IDH1 depleted D458 or D458IR MB cell lines. Total area (μM^2^) growth of neurospheres is shown, N=3, Mean ± SD. (Below) Scatter plot neurosphere size, mean ±SD. Each dot represents a neurosphere. T-test; **,p<0.01; ***,p<0.001; ****,p<0.0001. **D**. Log IC50 determination of IDH305, GSK321, and IDH1–13 on D458, D425 sensitive and D458IR, D425IR cell lines. N=3. Mean ± SEM. *See also* extended data Figure 5C. **E**. Box and whisker plot of α-Ketoglutarate assay on D458, D458IR, D425, D425IR cells treated with IDH305 (7μM) or GSK321 (1μM). T-test, **,p<0.01; *,p<0.05. **F**. Scatter dot plot of ALDH+ cells in D458, D458IR, D425, D425IR treated with DMSO or IDH305 (7μM). Mean ± SD. Two-way Anova; **,p<0.01; ***,p<0.001. **G**. Extreme limiting dilution assay in D458IR and D425IR treated with IDH305 (7μM) or GSK321 (1μM). Log-fraction plot of the limiting dilution model, where the slope of the line is the log-active cell fraction. Chi-square test; ***,p<0.001. **H**. Survival fraction plot. (Right) Survival enhancement ratio. N=3. Two-way Anova, **,p<0.01; ***,p<0.001. **I**. Model of citrate production from glutamine. 13C5, 15N2 L-glutamine is catabolized into α-ketoglutarate (C5) which contributes to citrate production through reductive carboxylation (orange arrows, C5) and oxidative metabolism (blue arrows, C4). **J**. Scatter bar plot of labeled 13C5, 15N2 L-Glut. 13C4, 13C5, and 13C6 are derived from labeled 13C5, 15N2 L-Glut. Total carbon is 12C. N=3. Mean ± SD. Two-way Anova, *,p<0.05; **,p<0.01; ***,p<0.001; ****,p<0.0001.

Several epigenetic modifiers utilize α-KG as a substrate for enzymatic activity. Isocitrate dehydrogenase (IDH1) is an essential enzyme in the conversion of citrate to α-KG, and glutamate to α-KG in the presence of hypoxia. Additionally, IDH1 is used to reduce ROS through the production of NADPH. An expanding progenitor_photoreceptor population in recurrent patient tumors and a reprogrammed progenitor population in murine resistance models suggests that IDH1 inhibition, a central participant in metabolic and epigenetic programs, may attenuate resistance. Thus, to evaluate the role of IDH1 in radiation resistance, radiation-resistant MYC-amplified MB cell lines, D458IR and D425IR, were generated. A global metabolomic analysis of D458/D458IR and D425/D425IR cells evaluated metabolic changes in radiation-resistant cells (Figure 6A, Extended Data Figure 4). The depletion of D-glucose, nucleic acids, and lactate indicated that radiation-resistant cells had a higher rate of metabolism, causing carbon sources to become rapidly exhausted and cells to behave as nutrient-deprived. The elevated levels of metabolites (citrate, acyl-co, ADP) affiliated with the TCA cycle, glutamate, and lipid metabolism align with the *in vivo* resistant models and recurrent patient samples, suggesting alternative carbon sources fueling cell growth (Figure 6A, B).

Next, *wtIDH1* expression was inhibited by shRNA knockdown under NSC conditions. The proliferation and self-renewal of neurospheres was significantly reduced, suggesting reduction of *IDH1* affects stem cell-like characteristics (Figure 6C). To assess whether therapeutic inhibition limited IR-resistant cell viability, three IDH1 inhibitors (IDH1*i*) were examined. IDH305 and GSK321 are selective brain-penetrant allosteric inhibitors designed to target the R132H mutation. However, both exhibit considerable activity against wild-type IDH1^33,34^, and inhibited growth of D458IR and D425IR lines at 7–10μM and 1μM, respectively (Figure 6D). The third inhibitor, IDH1–13, specifically inhibits wtIDH1, and reduced the viability of radiation resistant cells at 1.5 μM (Figure 6D, Extended Data Figure 4)^34^. Consideration was given to the mutant IDH1 inhibitor, Ivosidenib/AG-120, currently in phase I clinical trials for AML. However, AG-120 was ineffective in parent and radiation-resistant MB cells in both normal and NSC media (data not shown). Additionally, treatment with IDH305 or GSK321 at IC_50_ depleted α-KG production in D458IR by ~50%, and 40%−50% in D425IR cells (Figure 6E). Similar to *IDH1* KD, treatment with IDH1*i*’s reduced ALDH+ (stem-like marker) cells by 38% in D458 and D458IR cells, and by 60% in D425IR cells (Figure 6F). An extreme limiting dilution assay (ELDA) showed a 50% reduction in stem cell frequency in radiation-resistant and radiation-sensitive D458 and D425 cells upon IDH1*i* (Figure 6G, Extended Data Figure 4). Radiation-resistant cells exhibit an enhanced Survival Fraction (SF), requiring up to 6 Gy to reduce the same number of colonies as parental cells. Following treatment with IDH1*i,* the amount of radiation required to reduce 70% of colonies decreased to 4 Gy. Additionally, the sensitivity enhancement ratio significantly increased in the presence of IDH1 inhibitors (Figure 6H). These data suggest that IDH1 depletion reduces resilience against secondary radiation exposure.

Single-cell RNA-seq and scMultiome-Seq revealed a heterogeneous tumor with pockets of hypoxia and metabolically altered programming. Thus, to determine the effect of radiation resistance on metabolism, 13C5,15N2-labeled glutamine was used to track glutamine under NSC conditions (Figure 6I). Compared to the parent cells, radiation-resistant D458 cells showed an increase in total carbon (12C) incorporation for succinate, citrate, α-KG, aspartate, and glutathione levels; these were depleted upon IDH1 suppression. Levels of glutamate, α-KG, citrate, and aspartate labeling increased in radiation-resistant cells compared to sensitive (parent) cells (Figure 6J). Additionally, a significant decrease in citrate levels was observed, and nitrogen (15N1) levels were depleted in aspartate with IDH1i. This further suggested that inhibiting IDH1 blocks glutamate as an alternate carbon source specifically altering the availability of αKG and acetyl-coA.

### Epigenetic restructuring in radiation-resistant cells triggers bivalency at pluripotent gene promoters, which are partially restored to the parental state by IDH1 inhibition.

**Extended Data Figure 5.**
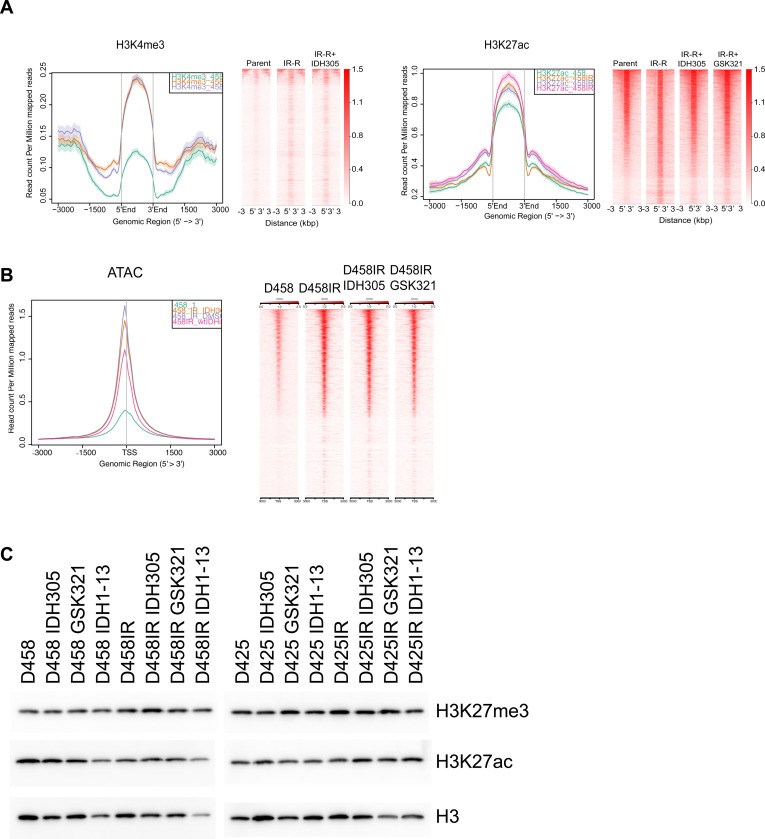
Global chromatin histone modifications remain unchanged despite shifting accessibility and H3K27ac deposits. **A.** H3K27ac and H3K4me3 enhancer region occupancy in D458 (parent), D458IR (IR-R), D458IR + IDH305, D458IR+GSK321. N=2. Enhancer heatmap shown on the right. **B**. ATAC-seq profile map, and corresponding heatmap. ATAC-seq was performed on D458, D458IR, D458IR + IDH305, D458IR+GSK321. N=2. **C**. Western blot on total H3K27me3, H3K27ac, and H3. D458, D458IR, D425, and D425IR cells were treated with DMSO, or IDH305 (7μM), GSK321 (1μM), IDH1–13 (1.5μM) for 48hrs, then histones were isolated. N=3.

**Figure 7.**
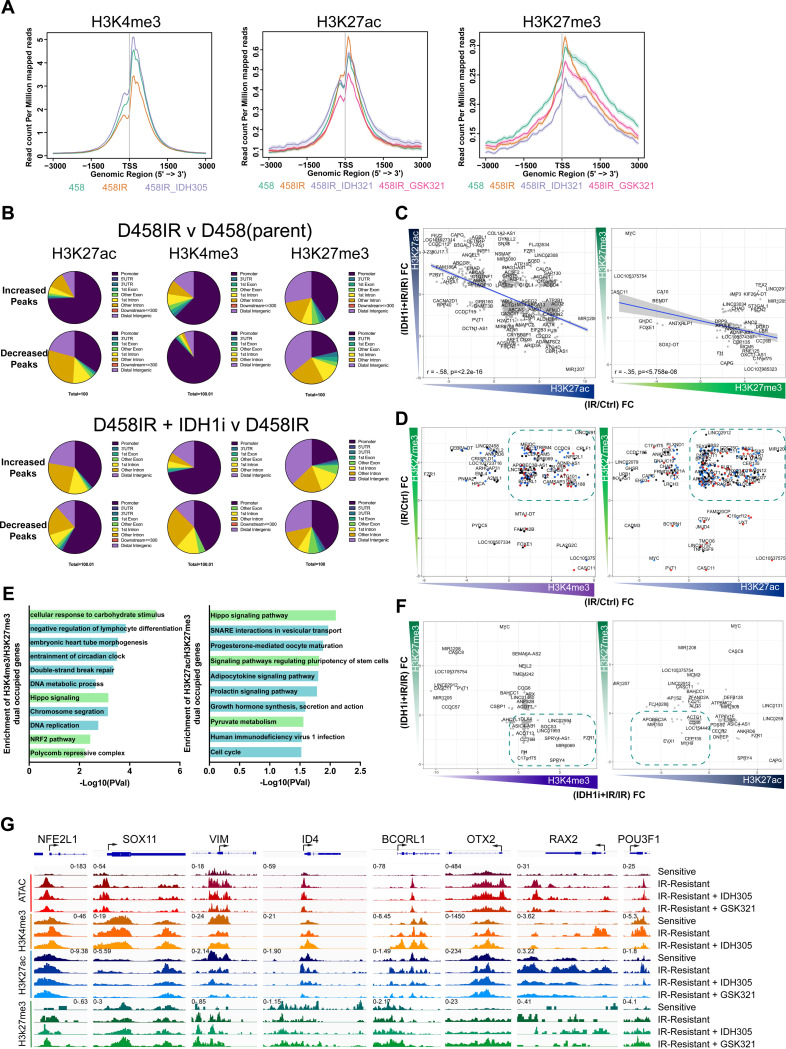
Restoration of parental epigenetic profile in radiation-resistant cells by IDH1 inhibition. **A.** Profile heatmap of H3K4me3, H3K27ac, and H3K27me3 −3Kb upstream and 3Kb downstream of the TSS. D458 (parent), D458IR were treated with IDH305 (7μM) or GSK321 (1μM) for 48 hours. **B.** Annotation of H3K27ac, H3K4me3, and H3K27me3 increased and decreased peak occupancy in D458IR(resistant) compared to D458(parent). (Below) Increased and decreased peak occupancy in D458IR(resistant) treated with IDH305 compared to DMSO treated D458IR. **C.** Scatter plots of D458IR/D458 Log_2_FC ratio compared to D458IR +IDH1i/D458IR for H3K27ac and H3K27me3. Pearson’s correlation score and P-value are shown. **D.** Scatter plots of D458IR/D458 Log_2_FC ratio compared to H3K4me3 compared to H3K27me3 and H3K27ac compared to H3K27me3. DEGs from scRNA-seq is overlayed on gene identified peaks, red genes up and blue genes downregulated. **E.** Enrichment analysis of dual occupied H3K4me3/H3K27me3 peaks and H3K27ac/H3K27me3 peaks. − Log10(Pval) is reported. **F.** Scatter plots of D458IR + IDH1i/D458IR Log_2_FC H3K4me3 compared to H3K27me3 and H3K27ac compared to H3K27me3. **G.** Genome browser view of representative gene loci from the progenitor_photoreceptor signature. Chromatin accessibility (ATAC-seq), H3K4me3, H3K27ac, H3K27me3 occupancy in D458 (sensitive), D458IR (IR-resistant), D458IR (IR-resistant) + IDH305 (7μM) or + GSK321 (1μM) are displayed. Scale for each histone mark is shown. All CUT and RUN experiments represent a N=2.

The utilization of glutamine in both glutaminolysis and glutamine decarboxylation enhances α-KG production and survival despite radiation damage. To understand the epigenetic effects of metabolic reprogramming, H3K4me3, H3K27ac, and H3K27me3 occupancy were assessed using CUT&RUN (Figure 7). Radiation-resistant cells exhibited depleted H3K4me3 deposits and enhanced H3K27ac and H3K27me3 peaks at promoter regions. Moreover, depletion of H3K27ac and H3K27me3 was observed in distal intergenic and intron regions (Figure 7A, B). However, IDH1 suppression restored global H3K4me3 levels, whereas H3K27ac and H3K27me3 levels were reduced below the parent cell line at the transcriptional start site (TSS; Figure 7A). Radiation-resistant cells treated with the IDH1*i* showed a reversal in the diminished peaks compared to the parent cell line. H3K4me3 peaks were enhanced in the promoter regions, and H3K27ac and H3K27me3 peaks were enhanced in the distal intergenic and intron regions (Figure 7B). Given the decrease in H3K27ac at intergenic regions, H3K27ac levels were evaluated at enhancer regions, revealing a global increase in H3K27ac levels at enhancers, which remained unchanged under IDH1 suppression (Extended Data Figure 5).

To understand whether the decreased gene-associated peaks in radiation-resistant cells were associated with the peaks enhanced by IDH1 inhibition, H3K27ac and H3K27me3 peaks in radiation-resistant cells were compared to those in radiation-resistant cells treated with an IDH1*i*. An inverse correlation between radiation-resistant and enhanced peaks after IDH1 inhibition suggested that the redistribution of H3K27ac and H3K27me3 peaks in radiation-resistant cells was partially recovered by IDH1 suppression (Figure 7C).

Bivalent promoters are commonly associated with regulation of pluripotent stem cell regulation but also contribute to cancer development^35–38^. To evaluate the overlap between H3K4me3 and H3K27me3, we mapped peaks and DEGs from scRNA-seq of resistant MB tumors generated *in vivo* (Figure 7D). Genes with enhanced H3K4me3 and depleted H3K27me3 levels were positively expressed, whereas those with enhanced H3K27me3 and depleted H3K4me3 levels were repressed. Considerable overlap between amplified H3K4me3 and H3K27me3 deposits was detected for up-and downregulated genes (Figure 7D). Similarly, H3K27ac and H3K27me3 overlap at promoter regions was also enhanced in radiation-resistant cells (Figure 7D). Gene ontology of the dual-occupied genes was examined to understand which gene-associated regions showed increased bivalency. H3K4me3 and H3K27me3 dual-occupied genes mediated glucose consumption (*SRF, SLC2A5*), TGFβ signaling (*ID2, LEFTY*), Hippo signaling (TEAD2), and the polycomb repressive complex (*BCORL1*; Figure 7E). Similarly, H3K27ac and H3K27me3 bivalency increased at the promoters of genes regulating stem cell pluripotency (*WNT9a* and *TBX3*), Hippo signaling (*TEAD2* and *BBC3*), and pyruvate metabolism (*FH* and *PC*) (Figure 7E). To understand the response to IDH1 suppression, the gene-associated peak location after IDH1 inhibition was compared. H3K27me3 peaks were reduced, whereas H3K4me3 peaks remained largely enhanced, excluding several key genes (*MYC, FZR1*, and *SPRY4*) which reverse the epigenetic profile (Figure 7F). Comparably, the fold-change after treatment showed a minimal decline and enhancement in both H3K27ac and H3K27me3 peaks (Figure 7F).

To determine whether bivalency was triggered by genes within the progenitor_photoreceptor gene signature, individual peak profiles were examined. ATAC-seq was conducted on parent, and radiation-resistant cells after exposure to an IDH1*i*. ATAC sequencing of resistant cells revealed an opening of chromatin regions, whereas IDH1 treatment diminished chromatin accessibility (Extended Data Figure 5). Comparison of ATAC and histone peak profiles of several genes in the progenitor_photoreceptor signature showed radiation-resistant cell amplification of H3K4me3 and H3K27ac for *NFE2L1, SOX11*, *ID4, RAX2*, and *POU3F1*. Similarly, H3K27me3 levels increased compared to sensitive (parent) cells; treatment with IDH1*i* reversed and/or diminished these effects (Figure 7G). These results suggested that radiation-resistance may be facilitated by a bivalency switch on pluripotent gene promoters mediating Hippo signaling, TGFβ signaling, and metabolism. However, this can be disrupted by IDH1 suppression reinstating the epigenetic profile of the parent cells. Suppression of IDH1 as a mechanistic key to supplying the substrates and energy source for this transition can impede epigenetic and metabolic reprogramming.

## Discussion

In the present study, the key differences in recurrent medulloblastoma compared to primary tumor sites were evaluated utilizing scMulti-omic sequencing. This enabled the characterization of the genetic and epigenetic intratumor heterogeneity with further attention placed on the development of radiation tolerance. Therapy tolerant cells (TTC) also called drug tolerant persister cells (DTP) are phenotypically and genomically heterogeneous^39^. DTP cells are generally characterized by quiescence and slow proliferation, altered metabolism, altered epigenetic programming, resistance to apoptosis and immune evasion^40^. The current study presents evidence Group 3 MB heterogeneity arises from differential lineage commitment controlled by distinct transcription factor_driven gene regulatory networks (TF-GRNs). These are altered in therapy-resistant tumors, resulting in an enrichment of DTP cells mediated by a neural progenitor_photoreceptor GRN. The single-cell ATAC seq data further showed that the chromatin of therapy tolerant medulloblastoma tumors was restructured to exhibit increased accessibility, particularly at cis-regulatory elements associated with regulating chromatin silencing and metabolic programs. Underlying these features is increased cellular plasticity that rewires cellular signaling to evade cytotoxic therapies. These data show that as the primary tumor progresses to recurrence, the early progenitors undergo a cell-state change, further amplifying a multipotent state of early photoreceptor-like progenitor tumor cells.

To more distinctly resolve the contribution of radiation resistance to recurrence, an *in vivo* model of radiation tolerance was developed. The results showed a rewiring from differentiation programs and cell fate commitment to regulatory networks controlling metabolic programs mediating stem cell differentiation in radiation-resistant tumors. GRNs associated with cellular respiration and α-KG metabolism were enriched in the resistant tumors, particularly in the progenitor_photoreceptor population. Metabolomics confirmed that radiation-resistant cells had a higher rate of metabolism with enriched metabolites associated with the TCA cycle and glutamate metabolism. Critically, wtIDH1 enzymatically regulates energy production and glutamine metabolism while producing α-KG, a crucial substrate for iron-dependent enzymes involved in epigenetic programming and maintaining of pluripotent embryonic stem cells^41–43^. Inhibition of wtIDH1 reversed radiation resistance and depletion of a stem cell state in IR-resistant MB cells. Furthermore, chromatin was remodeled during radiation resistance, a phenomenon that can be reversed upon wtIDH1 inhibition. This provides additional evidence supporting the assertion that epigenetic reprogramming plays a crucial role in driving therapy resistance.

Collectively, thus study links the persistence of the progenitor_photoreceptor GRN within the radiation-resistance model and recurrent patient tumors to the rewiring of epigenetically defined_pluripotency programs via IDH1-mediated metabolic reprogramming. These data provide evidence that targeting wtIDH1 in relapsed MB maybe an effective therapeutic approach for this complex tumor.

## Methods

### scMultiome sample preparation.

*Patient samples*: Human MB samples were collected and viably banked at the time of surgery at Children’s Colorado with consent (COM-IRB 95–500) over a 10-year period. Samples were processed to obtain single nuclei for single-cell Multiome sequencing according to the 10X Genomics (Pleasanton, CA) protocol. Briefly, a rice grain size of cyropreserved tumor tissue was homogenized in chilled 0.1X Lysis Buffer with a pellet pestle. Lysis buffer(10mM Tris-HCl (pH 7.4), 10mM NaCl, 3mM MgCl_2_, 0.1%Tween-20, 0.1% NP-40, 0.01% Digitonin, 1% BSA, 1mM DTT, 1U/μL RNase Inhibitor) and Lysis Dilution buffer(10mM Tris-HCl(pH7.4), 10mM NaCl, 3mM MgCl_2_, 1%BSA, 1mM DTT, 1U/μL RNAse inhibitor). Samples were incubated on ice for 5 min, mixed, then iced again 10 min. Samples are washed with 500μL of chilled wash buffer (10mM Tris-HCl(pH7.4), 10mM NaCl, 3mM MgCl_2_, 1%BSA, 0.1% Tween-20, 1mM DTT, 1U/μL RNase inhibitor) and passed through a 40μM strainer twice. The samples were centrifuged at 500 rcf for 5 min at 4°C. The nuclei pellet was then washed twice with 1 mL of chilled wash buffer. Nuclei were counted on the Countess II FL automated cell counter, and nuclei were diluted in nuclei buffer and submitted for sequencing. The Chromium Next GEM Chip J Single Cell Kit (10xGenomics), Dual Index Kit TT set A, and Chromium Next GEM Single Cell Multiome ATAC + Gene Expression Reagent Bundle were used for sample preparation and library sequencing on the Chromium *X.* *PDX model:* MED411 cell isolation from mouse xenograft was conducted with NeuroCult^™^ Enzymatic Dissociation Kit (STEMCELL Technologies). Mouse cells were depleted from the single cell suspension with the Mouse Cell Depletion Kit on LS Columns (Miltenyi Biotec). Isolated human cells were then processed according to 10X Genomics nuclei isolation protocol for single-cell Multiome sequencing as described.

### scMultiome data processing.

The feature-barcode matrix was generated using cellranger-arc (10x Genomics, v2.0.2), aligning the sequenced reads to the human reference genome (10x Cell Ranger reference GRCh38 2020-A-2.0.0) followed by standard procedure using Seurat R package (v4.4.0). Cells with fewer than 200 genes detected were filtered from the scMultiome dataset. Low-quality cells with mapped reads in the mitochondrial genes >10% were removed. Doublets were identified using scDblFinder (v1.16.0). To address the issue of technical differences across samples, the cell count matrix was individually normalized by the total read count and log-transformed, and the top 2000 variable genes were selected for each sample using option “vst” in Seurat’s FindVariableFeatures function. Principal components analysis (PCA) was conducted on the scaled expression matrix. The top 15 principal components (PCs) were used to compute SharedNearestNeighbors (SNNs) in Seurat R package, and for UMAP embedding. Nucleosome signal was computed with Signac’s Nucleosome Signal function. Low-quality cells were removed from the scMultiome dataset based on the following criteria: < 2000 or > 100,000 fragments mapped to peak regions, nucleosome signal > 2, and TSS enrichment <1. MACS2 was employed to recall peaks for each sample. LSI was performed by Signac (v1.10.0). A joint neighbor graph that represents both gene expression and DNA accessibility measurements was done by FindMultiModalNeighbors function in Signac. UMAP was generated PCA dimensional reduction for gene expression and LSI indexing for DNA accessibility.

### Cell type Annotation.

Cells were clustered using FindClusters in Seurat with resolution of 0.5. Clusters were annotated based on known marker genes and integrated datasets^23–30^.

### Trajectory analysis.

Cell trajectories were analyzed using Monocle2 (v2.28.0). Selected cells were first down-sampled to a total of 5,000 to remove trends driven by different cell abundance in different cell types. The dispersion table function in Moncle2 was applied to calculate gene variances. Top genes with mean expression > 0.1 were selected for further analysis. UMAP creation and pseudotime estimation were performed by the reduceDimension and orderCells functions in Monocle2, respectively. Branched expression analysis modeling (BEAM) was utilized to identify DEGs along pseudotime in different lineages.

### Transcriptional factor analysis.

pySCENIC (v0.12.1) was used to infer transcription factors. Genes co-expressed with transcription factors were identified using GENIE3. AUCell was then used to score the activity of each regulon in each cell. Regulons with the highest activity in each group were identified by the Wilcox rank-sum test followed by Bonferroni correction with an adjusted *P-value* < 0.01.

### scRNA sequencing.

D458 xenograft tumors were harvested from the cerebellum of mice. Tumors were chopped with razor blades and dissociated in RPMI media. Live cell suspensions were flow sorted based on green fluorescent protein (GFP) expression on a MoFlo XDP-Flex (Beckman Coulter) and 2,000/μL GFP+ cells were submitted to the University of Colorado Genomics core for scRNA sequencing with the Chromium X. N=6

### scRNA-sequencing data processing.

Data was processed using nf-core/scrnaseq v2.4.0 (doi: 10.5281/zenodo.3568187) of the nf-core collection of workflows^44^, utilizing reproducible software environments from the Bioconda^45^ and Biocontainers^46^ projects. *Analysis of single-cell/single-nuclei RNA-Seq data from MED411 PDX model:* Data integration, clustering analysis, and plotting were performed in Seurat (5.0.0), running under R version 4.3.1. Single-sample gene set enrichment analysis (ssGSEA, version 10.1.0) was performed using GenePattern (Broad Institute). Integration and analysis of scRNA-seq data: The gene expression matrix file for scRNA-Seq performed on MED411 patient samples was obtained from Gene Expression Omnibus archive GSE119926^14^. These data were merged with the GEX single nuclei gene expression data from mouse PDX samples 914 – 918, which were derived from MED411. Data were log-normalized, and SCTransform (v2) was performed in Seurat using percent mitochondrial gene expression as the regression variable. Harmony integration^47^ was applied but deemed unnecessary, presumably because all samples originated from a single patient. Dataset integration was validated by comparing Uniform Manifold and Projection (UMAP) plots of the patient sample and the untreated PDX control. To generate the clustering analysis, a principal component analysis (PCA) with 30 dimensions was used to generate the UMAP projection. FindClusters was run in Seurat with resolution of 0.6. Differential expression analysis was performed on the SCT assay using FindAllMarkers with min.pct and logfc threshold set to 0.25. Only positive differential expression (enrichment) was identified. The progenitor_photoreceptor gene signature was compared between the RT and control using ModuleScore and the following genes: (“*SOX4”, “TEAD1”, “HSPH1”, “KDM5B”, “KDM4B”, “NFE2L1”, “EVX1”, “SOX11”, “RCOR1”, “BHLHE40”, “CRX*”). ssGSEA analysis: Aggregate expression was computed by gene for each sample. The aggregate data were normalized using the calcNormFactors function of EdgeR with the trimmed mean of M-values “TMM” method. Data were output without log transformation. ssGSEA was performed using GenePattern with no sample normalization and a weighting exponent of 0.75. Output gct files from ssGSEA were filtered, and heatmaps were plotted using pheatmap (1.0.12) in R.

### Cell culture.

D425 (RRID:CVCL_1275; male) and D458 (RRID:CVCL_1161; male) cell lines were kindly provided by Dr. Darell D. Bigner (Duke University Medical Center, NC). Cells were cultured in Dulbecco’s modified eagle’s medium (DMEM) supplemented with 10% FBS (Sigma-Aldrich), 1 mM sodium pyruvate (Gibco), 1× penicillin/streptomycin solution (Cellgro), and with 1× L-glutamine (Cellgro). NHA cells were provided by Cynthia Hawkins (University of Toronto Hospital for Sick Children). MED411FH was originally harvested from a large cell anaplastic MB that was molecularly characterized as a Group 3 tumor. All cell lines were cultured at 37 °C with 95% air and 5% CO2. Cell lines were authenticated with STR fingerprinting using the Globalfiler^®^ System (Thermo Fisher Scientific) and processed on ABI 3500Xl Genetic Analyzer, and mycoplasma testing was conducted with the Venor^™^ GeM Mycoplasma Detection Kit (Cat# MP0025–1kt, Sigma-Aldrich) both were done as recent as 2/23/2024. All cell lines were maintained for a maximum of 20 passages for the duration of experiments.

### Animal model.

Female athymic Nude-Foxn1^nu^ mice aged 4 to 8 weeks and female NOD-scid gamma (NSG^™^) aged 4 to 8 weeks were utilized for orthotopic xenograft studies. Mice were housed in ventilated cages with appropriate nesting enrichments, bedding, food and water according to the University of Colorado regulations. For the D458 resistant study, cells were collected and resuspended as a single cell suspension of 20000 cells/3 μL in serum free media. MED411 cells were injected at 200,000/ 3 μL in serum-free media. Intracranial injection of cells into outbred athymic Nude-Foxn1^nu^ mice (Jackson labs strain 07850) or NSG (Jackson labs strain 005557) was performed at 1.5mm lateral and 2mm posterior of lambda at 400 nanoliters/min. Mice were monitored for tumor growth daily and euthanized by asphyxiation and cervical dislocation when 15% weight loss was reached or signs of tumor (hunched back, paralysis, or tilted head) were evident. Littermates were randomly assigned to experimental groups. All animal procedures were performed in accordance with the National Research Council’s Guide for the Care and Use of Laboratory Animals and were approved by the University of Colorado Anschutz Medical Campus Institutional Animal Care and Use Committee.

### Irradiation of animals.

Animal radiotherapy was performed using the X-Rad SmART small animal irradiator (Precision X-Ray, North Branford CT). Under isoflurane anesthesia, mice received 2.5Gy to the cerebellum, in five fractions of 0.5Gy, delivered on consecutive days with pairs of 10mm-diameter lateral beams.

### Viral transduction.

*IDH1* stable knockdown cell lines were established using gene-specific mission shRNAs (Cat# TRCN0000027249, TRCN0000027253) or a non-targeting control (Sigma-Aldrich). HEK293FT cells were plated at 5×10^6^ cells/10cm dish 1 day before transfection in complete media (DMEM, 10%FBS, 5% penicillin and streptomycin). Lipofectamine 3000 (Thermo Fisher Scientific, Cat#L3000008) was diluted in Opti-MEM at a ratio of 1:16.6. P3000 was diluted at 1:25 in Opti-MEM along with 1μg of pMD2.G, 2 μg of psPAX2, and 1.2 μg of pLenti-shIDH1 expression vector. Both mixes were combined and incubated for 15 mins at 25°C. The mixture was added dropwise to HEK293FT cells in fresh media, 24 hours later, the media was replaced with DMEM/2.5% FBS. Viral particles were collected at 48 hours in a 15 mL conical and centrifuged for 5 min at 500xg. Viruses were filtered through a 0.45 μm filter and stored in 1 mL aliquots at −80. To generate the stable knockdown cell lines, cells were plated at 5×10^5^ in a 6-well dish in growth medium containing 8μg/mL polybrene. Viral particles were added with 8μg/mL polybrene and incubated for 7 hrs. Transduced cells were disaggregated, cultured, and selected with 2μg/mL puromycin for 14 days. Transformants were confirmed by qRT-PCR and western blot.

### Compounds.

IDH305 (HY-104036), IDH1 inhibitor 2 (compound 13) (HY-128661), GSK321 (HY-18888) were all obtained from MedChemExpress. Chemical compounds were diluted in dimethylsulfoxide DMSO at 10mM stock concentration. Working concentrations were then diluted in serum-free media.

### Survival Enhancement Ratio and Methylcellulose assay.

D458, D458IR, D425, and D425IR cells were exposed to irradiation with the Precision MultiRad350. Dosages included 0, 2, 4, 6,and 8Gy. 48 hours after exposure 1000 up to 10,000 cells/3mL were plated in a 1:1 mixture of 2.6% methylcellulose and complete growth medium. Cells were allowed to grow for 10 days; colonies were stained with nitrotetrazolium blue chloride (Sigma-Aldrich) at 1.5mg/mL in PBS for 24hrs at 37°C and counted.

### Extreme limiting dilution assay.

Cells were seeded in a 96-well ultra-low-attachment round-bottom tissue culture plate in serum-free media at 1 cell/well, 10 cells/well, and 100 cells/well. Cells were allowed to grow for 14 days, then the number neurosphere containing wells were counted. ELDA software (http://bioinf.wehi.edu.au/software/elda/) was used to calculate the comparative self-renewal potential of cells^48^.

### Neurosphere growth assay.

D458IR shNull, D458IR shIDH1, D425IR shNull, D425IR shIDH1 D458 or D425 shNull and shCDK7 cells were serially diluted at 100,10, and single-cell suspensions in neurosphere growth media (neurobasal medium, B-27+vitamin A, L-glutamine, pen/strep, epidermal growth factor (EGF), and fibroblast growth factor (FGF). Cells were grown for 14 days with media replacement every 3 days at 37°C. At 14 days, spheres were collected, disassociated, and replated at 100, 10, and single-cell suspensions; thereafter, spheres were allowed to grow for an additional 14 days. Neurosphere proliferation and real-time monitoring were performed using the Incucyte^®^ S3 live cell imaging system (Essen Bioscience).

### α-Ketoglutarate assay.

D458, D458IR, D425, and D425IR cells were treated for 48 hours with IDH305 or GSK321 at IC50 or DMSO. α-KG standards were prepared at 0, 2, 4, 6, 8, and 10 nmole/well in 50μL of α-KG assay buffer. Cells were collected and resuspended in 100μL of ice-cold α-KG buffer. 50μL of sample was mixed with 50 μL of reaction mix and incubated at 37°C in the dark for 30mins as per the manufacturer’s instructions (Sigma Aldrich, α-Ketoglutarate Quantitation Kit, Cat#MAK541). Absorbance was measured at 570nm.

### ALDEFLUOR^™^ Assay.

D458, D458IR, D425, and D425IR cells were treated for 48 hours with IDH305 at IC50 or DMSO. Cells were collected and then diluted at 1×10^6^/mL in ALDEFLUOUR assay buffer. Cells were exposed to 5μL of ALDEFLUOR reagent/mL for 60 mins at 37°C as per the manufacturer’s instructions (STEMCELL technologies, ALDEFLUOR^™^ Kit (Cat#01700)). Samples were counterstained with Draq5^™^ (eBioscience^™^). Following incubation, samples were assayed on the CytoFLEX Flow Cytometer. 10000 events were collected. Gating and analysis were performed using FlowJo Software.

### IC50 determination.

Cells were plated at 1000 cells/well in a 96-well dish and exposed to DMSO, IDH305, GSK321, or IDH1–13 for 48hrs at 0, 0.1, 0.25, 0.5, 1, 2, 5, 7, 10, 20, and 30μM. Cell viability was assessed with the CellTiter 96^®^ Aqueous One Solution Cell Proliferation Assay #G3580 (Promega) as per the manufacturer’s instructions.

### Mass spectrometry and 13C5, 15N2 L-Glutamine Trace.

D458 (Sensitive) and D458IR (IR-resistant) cells were plated at 1×10^6^ in NSA media with DMSO or 7μM IDH305. At 24h NSA media was replaced with glutamine-deficient NSA media + 13C5, 15N2 L-glutamine (0.5mM) + 7μM IDH305 or DMSO. Cell pellets were collected after 4 h, and samples were snap-frozen. For high-throughput polar metabolite profiling, D458, D458IR, D425, and D425IR cell pellets were collected and snap-frozen. Samples were submitted to the University of Colorado School of Medicine Metabolomics Core for analysis. Metabolites were extracted using 5:3:2 methanol:acetonitrile:water and profiled using a Thermo Vanquish UHPLC coupled to a Thermo Orbitrap Exploris 120 mass spectrometer as previously described^49^. Statistical and enrichment analyses on metabolite profiles were conducted using Metaboanalyst^50^. Flux analysis was performed by the University of Colorado School of Medicine Metabolomics core.

### ATAC-Sequencing.

D458 and D458IR cells were exposed to IDH305 or GSK321 at IC50 for 48h. 50,000 cells were collected and washed in cold 1x PBS. Cell pellets were resuspended in 450μL of cold hypotonic buffer (10mM Tris-HCL, pH7.4, 10mM NaCl, 3mM MgCl_2_). Next, 50μL of 1% IGEPAL CA-630 was added and inverted to mix. Samples were iced for 10min, centrifuged for 10mins at 4°C. Cells pellets were resuspended in transposition reaction mix and incubated at 37°C for 60min. Following transposition, the DNA was isolated, purified, and prepped for sequencing. The sequencing library is purified with AMpure XP beads (Millipore). ATAC assay kit (Cell Biologics^™^ ATAC-Seq Kit (Cat# CB6936)).

### Cut and Run-Sequencing.

D458 and D458IR were exposed to 10μM 1DH305, or 1μM GSK321 for 48hrs; 5×10^5^ cells were collected for analysis by CUT&RUN (EpiCypher, CUTANA^™^, CUT&RUN Kit Version 4 #14–1048) per the manufacturer’s instructions. Antibodies against, α-H3K4me3 and IgG controls were validated antibodies from EpiCypher. α-H3K27me3 (Cell Signaling technologies, #9733) and α-H3K27ac (Cell signaling technologies, #8173). CUT&RUN library prep was performed with the NEBNext^®^Ultra^™^II DNA Library Prep Kit for Illumina^®^ (NEB #E7645L) and NEBNext Multiplex Oligos for Illumina (NEB#E7350). Sequencing was performed on a Novaseq 6000. CUT&RUN-seq reads were aligned to the reference human genome hg38 using BOWTIE (v.2.3.4.1). Aligned reads were stripped of duplicate reads using Sambamba (v.0.6.8). Peaks were called using the program MACS (v2.1.2), with the narrow peak mode using matched input controls and a q-value of 0.00001. Peaks in the blacklisted genomic regions identified by the ENCODE consortium were excluded using bedtools. For downstream analysis and visualization, bamCoverage was used to generate bigwig files and density maps were produced using IGV tools. Group 3 MB enhancers were defined based on H3K27ac signals. Regions within 1kb of RefSeq TSS locations and peaks with strong H3K4me3 signals typical of active promoters were subtracted from these signals. Annotation and visualization of the peaks were conducted using ChIPseeker (v3.18). Differentially marked genes were calculated using DiffBind and DESeq2, based on the threshold of FDR < 0.05 and FC ≥ 2.

### Statistical analysis.

All experiments were performed with at least three independent replications. All data was collected in Excel, and Graphpad Prism 10.3.1 statistical software was used for analysis. Data are presented as the mean ± standard deviation (SD). *P-values* <0.05 were considered significant, where p-value<0.05 (*), p-value<0.01(**), p-value<0.001 (***), p-value<0.0001 (****). Unpaired, two-tailed, t-tests were used for two-group comparisons, and ANOVA analysis and Dunnett’s test was performed for multiple group comparisons. Patient statistical analyses were performed using Wilcox rank-sum test. Multiple comparison corrections were applied using the Bonferroni method.

## Extended Data

## Data Availability

All sequencing files used for this study are available through GEO under the submission series (). The Hovestadt sequencing dataset was obtained from Gene Expression Omnibus (GSE119926). Okonechnikov^32^ and Cavalli^3^ datasets were accessed through R2: Genomics Analysis and Visualization Platform (http://r2.amc.nl)’.
